# *Notes from the Field:* COVID-19 Vaccination Coverage Among Persons Experiencing Homelessness — Six U.S. Jurisdictions, December 2020–August 2021

**DOI:** 10.15585/mmwr.mm7048a4

**Published:** 2021-12-03

**Authors:** Martha P. Montgomery, Ashley A. Meehan, Antea Cooper, Karrie-Ann Toews, Isaac Ghinai, Mary Kate Schroeter, Rachael Gibbs, Najibah Rehman, Katerina S. Stylianou, David Yeh, Nikki Thomas-Campbell, Nathalie C. Washington, Hannah K. Brosnan, Alicia H. Chang, Ayodele Gomih, Cathy Ngo, Katherine Diaz Vickery, Blair Harrison, Tyler N.A. Winkelman, Adam Gerstenfeld, Laura Zeilinger, Emily Mosites

**Affiliations:** ^1^CDC COVID-19 Response Team; ^2^Chicago Department of Public Health, Chicago, Illinois; ^3^Detroit Health Department, Detroit, Michigan; ^4^Fairfax County Department of Housing and Community Development, Fairfax, Virginia; ^5^Fairfax County Health Department, Fairfax, Virginia; ^6^COVID-19 Response, Acute Communicable Disease Control Program, Los Angeles County Department of Public Health, Los Angeles, California; ^7^COVID-19 Response, Tuberculosis Control Program, Los Angeles County Department of Public Health, Los Angeles, California; ^8^Health, Homelessness, and Criminal Justice Lab, Hennepin Healthcare Research Institute, Minneapolis, Minnesota; ^9^Hennepin County Health Care for the Homeless, Hennepin County Public Health, Minneapolis, Minnesota; ^10^Minnesota Department of Health; ^11^District of Columbia Department of Human Services, Washington, D.C.

COVID-19 outbreaks have been reported in homeless shelters across the United States (*1*). Many persons experiencing homelessness are older adults or persons with underlying medical conditions, placing them at increased risk for severe COVID-19–associated illness. The proportion of persons experiencing homelessness who are fully vaccinated against COVID-19 in the United States is currently unknown. Many persons experiencing homelessness express a willingness to receive the COVID-19 vaccine (*2*,*3*).

Through conversations with public health and housing assistance partners, CDC identified six* urban public health jurisdictions with data on vaccination coverage among persons experiencing homelessness. These six jurisdictions reported data on COVID-19 vaccinations^†^ administered to persons experiencing intermittent homelessness during December 13, 2020–August 31, 2021. Full vaccination status^§^ and evidence of coverage with at least 1 COVID-19 vaccine dose^¶^ among persons experiencing homelessness were obtained by performing data linkage between immunization information systems and homeless services data systems or through data collection during vaccination events at homeless service sites. Total populations of persons experiencing homelessness were estimated using either the total number of persons accessing homeless services during the study period or an annual census of persons experiencing homelessness.** Vaccination coverage and size of the general population in each jurisdiction were obtained from CDC’s COVID Data Tracker^††^ or from local health departments. The percentage point differences in vaccination coverage between persons experiencing homelessness and the general population were calculated, along with 95% CIs. This activity was reviewed by CDC and was conducted consistent with applicable federal law and CDC policy.^§§^

Full COVID-19 vaccination coverage among persons experiencing homelessness ranged from 18.6% to 44.5% in the six jurisdictions compared with 43.6% to 59.8% in the general population in each jurisdiction or corresponding area (Table). In each jurisdiction, full vaccination coverage among persons experiencing homelessness was substantially lower (11.2–37.2 percentage points) than that among the general population of the respective jurisdiction. Coverage with at least 1 COVID-19 vaccine dose across the six jurisdictions ranged from 22.0% to 52.0% among persons experiencing homelessness, and from 46.5% to 65.7% in the respective general populations.

**TABLE:** COVID-19 vaccination coverage among persons experiencing homelessness and the general population — six U.S. jurisdictions,* December 2020–August 2021

Characteristic	Jurisdiction (corresponding area for general population)					
	Chicago, Illinois	Detroit, Michigan (Wayne County)	Fairfax, Virginia (Fairfax County, Falls Church City, Fairfax City)	Los Angeles County, California	Hennepin County, Minnesota	District of Columbia
Earliest date of available data	Dec 13, 2020	Dec 19, 2020	Jan 25, 2021	Dec 15, 2020	May 1, 2021	Jan 29, 2021
Latest date of available data	Aug 31, 2021	Aug 30, 2021	Jul 31, 2021	Jul 31, 2021	Jul 31, 2021	Jul 31, 2021
Date of vaccine eligibility for persons experiencing homelessness	Jan 20, 2021	Jan 14, 2021	Jan 25, 2021	Mar 15, 2021	Jan 15, 2021	Jan 29, 2021
**Estimated population size**						
Persons experiencing homelessness, no.^†^	4,477	5,118	1,859	66,436	7,635	6,381
General population, no.^§^	2,693,959	1,749,343	1,183,521	10,039,107	1,265,843	705,749
**Fully vaccinated** ^¶^						
Persons experiencing homelessness,** no. (%)	1,993 (44.5)	950 (18.6)	465 (25.0)	23,353 (35.2)	1,712 (22.4)	1,265 (19.8)
General population,^††^ no. (%)	1,500,931 (55.7)	762,637 (43.6)	707,528 (59.8)	5,375,111 (53.5)	754,489 (59.6)	386,475 (54.8)
Difference (95% CI)	11.2 (9.7–12.7)	25.0 (23.9–26.1)	34.8 (32.8–36.8)	18.4 (18.0–18.8)	37.2 (36.2–38.1)	34.9 (33.9–35.9)
**≥1 dose** ^§§^						
Persons experiencing homelessness,** no. (%)	2,326 (52.0)	1,337 (26.1)	557 (30.0)	29,412 (44.3)	2,184 (28.6)	1,407 (22.0)
General population,^††^ no (%)	1,642,339 (61.0)	814,140 (46.5)	777,970 (65.7)	6,225,192 (62.0)	820,182 (64.8)	432,833 (61.3)
Percentage point difference^¶¶^ (95% CI)	9.0 (7.5–10.5)	20.4 (19.2–21.6)	35.8 (33.6–37.9)	17.7 (17.4–18.1)	36.2 (35.2–37.2)	39.3 (38.2–40.3)

* The six jurisdictions included Chicago, Illinois; Detroit, Michigan; Fairfax, Virginia; Los Angeles County, California; Hennepin County, Minnesota; and the District of Columbia. Most jurisdictions included persons living sheltered and those living unsheltered.

^†^ Population sizes for persons experiencing homelessness (all ages) were estimated using entry and exit dates in homeless service access data (Detroit, Fairfax, and Hennepin County), point-in-time count estimates (Chicago and Los Angeles), or both (District of Columbia).

^§^ Population sizes for the general population (all ages) for each jurisdiction or corresponding area were obtained from National Census Population Estimates from the 2019 Vintage U.S. Census Bureau Annual Estimates of the Resident Population for the United States: https://www2.census.gov/programs-surveys/popest/datasets/2010-2019/counties/totals/ (Accessed August 31, 2021). Population size for Chicago was obtained from the 2019 1-year American Community Survey estimate: https://www.census.gov/data/developers/data-sets/acs-1year.html (Accessed August 31, 2021). Population size for Fairfax was obtained from 2019 5-year American Community Survey estimate: https://www.census.gov/data/developers/data-sets/acs-5year.html (Accessed August 31, 2021).

^¶^ Fully vaccinated persons (homeless and general population) includes all persons who received 2 doses on different days (regardless of interval between doses) of the 2-dose mRNA series or received 1 dose of a single-dose vaccine and were at least 14 days after completion.

** Numbers of vaccinated persons experiencing homelessness were identified by performing record matching between immunization information systems and homeless service access data (Detroit and Hennepin County), using health care provider reports of vaccinations for persons experiencing homelessness (Chicago), or both (Fairfax, Los Angeles, and District of Columbia).

^††^ Numbers of vaccinated persons in the general population were obtained from CDC’s COVID Data Tracker: https://covid.cdc.gov/covid-data-tracker/#datatracker-home (Accessed August 31, 2021). Numbers of vaccinated persons in the general population for Chicago and Fairfax were obtained from the respective public health departments. Vaccinated persons included all persons in the jurisdiction from December 13, 2020, through the stated end date.

^§§^ Includes all persons who received at least 1 dose of COVID-19 vaccine, including those who received 1 dose of the single-dose vaccine.

^¶¶^ Between persons experiencing homelessness and the general population.

These estimates highlight relatively low COVID-19 vaccination coverage among persons experiencing homelessness compared with coverage in the general populations in a convenience sample of six jurisdictions. Estimating vaccination coverage for persons experiencing homelessness is challenging because housing status is not routinely collected in vaccination records. In addition, because homelessness could be temporary, estimating population size is difficult. Some health departments have overcome these challenges by fostering relationships with health clinics and homeless service providers. The use of integrated data systems to link deidentified, individual-level records across housing, health care, and public health systems is an emerging potential solution.

The findings in this report are subject to at least three limitations. First, because of varying data collection methods, comparison across jurisdictions was not possible. Second, the systems used for estimating homelessness rely on use of homeless services, and not all persons experiencing homelessness access these services, particularly persons living unsheltered. Finally, because of nonrandom selection and inclusion of only six jurisdictions, these findings are not generalizable to all persons experiencing homelessness in the United States, particularly in rural areas.

Given low COVID-19 vaccination coverage and increased risk for infection with SARS-CoV-2, the virus that causes COVID-19, in congregate settings (*4*), it is important that state and local health departments continue to follow CDC guidance to plan and respond to COVID-19 among persons experiencing homelessness.^¶¶ ^Vaccine access for persons experiencing homelessness can be enhanced by using multiple strategies (*5*), including pop-up vaccination clinics in convenient locations, mobile clinics in partnership with trusted providers, and street outreach teams. COVID-19 vaccination coverage can be improved by strengthening partnerships across health departments, health care clinics, and homeless service providers. Furthermore, including persons who have experienced homelessness in vaccination planning is critical to helping ensure approaches are tailored to the needs of persons experiencing homelessness.

## Footnotes

* The six jurisdictions included Chicago, Illinois; Detroit, Michigan; Fairfax, Virginia; Los Angeles County, California; Hennepin County, Minnesota; and the District of Columbia. Most jurisdictions included persons living sheltered and those living unsheltered.

† On December 12, 2020, the Advisory Committee on Immunization Practices issued an interim recommendation for the use of a vaccine for the prevention of COVID-19 in persons aged ≥16 years.

§ Fully vaccinated persons included those who received 2 doses on different days (regardless of time interval) of the 2-dose mRNA series or received 1 dose of a single-dose vaccine, at least 14 days earlier.

¶ Coverage with at least 1 dose included all persons who received at least 1 dose of the 2-dose mRNA series COVID-19 vaccine or those who received 1 dose of the single-dose vaccine.

**
https://www.ncbi.nlm.nih.gov/books/NBK519593/

†† https://data.cdc.gov/Vaccinations/COVID-19-Vaccinations-in-the-United-States-County/8xkx-amqh (Accessed August 31, 2021).

§§ 45 C.F.R. part 46.102(l)(2), 21 C.F.R. part 56; 42 U.S.C. Sect. 241(d); 5 U.S.C. Sect. 552a; 44 U.S.C. Sect. 3501 et seq.

¶¶ https://www.cdc.gov/coronavirus/2019-ncov/community/homeless-shelters/plan-prepare-respond.html (Accessed April 1, 2021).
